# Seeing what you want to see: priors for one's own actions represent exaggerated expectations of success

**DOI:** 10.3389/fnbeh.2014.00232

**Published:** 2014-06-27

**Authors:** Noham Wolpe, Daniel M. Wolpert, James B. Rowe

**Affiliations:** ^1^Department of Clinical Neurosciences, University of CambridgeCambridge, UK; ^2^Medical Research Council Cognition and Brain Sciences UnitCambridge, UK; ^3^Computational and Biological Learning Laboratory, Department of Engineering, University of CambridgeCambridge, UK

**Keywords:** voluntary action, sense of agency, illusions of superiority, goal-directed action, action observation, Bayesian, visual perception, sensorimotor prediction

## Abstract

People perceive the consequences of their own actions differently to how they perceive other sensory events. A large body of psychology research has shown that people also consistently overrate their own performance relative to others, yet little is known about how these “illusions of superiority” are normally maintained. Here we examined the visual perception of the sensory consequences of self-generated and observed goal-directed actions. Across a series of visuomotor tasks, we found that the perception of the sensory consequences of one's own actions is more biased toward success relative to the perception of observed actions. Using Bayesian models, we show that this bias could be explained by priors that represent exaggerated predictions of success. The degree of exaggeration of priors was unaffected by learning, but was correlated with individual differences in trait optimism. In contrast, when observing these actions, priors represented more accurate predictions of the actual performance. The results suggest that the brain internally represents optimistic predictions for one's own actions. Such exaggerated predictions bind the sensory consequences of our own actions with our intended goal, explaining how it is that when acting we tend to see what we want to see.

## General introduction

People perceive the sensory consequences of their own actions differently from similar sensory events that are externally caused. For example, the sensory effect of one's own action is typically perceived to occur earlier in time (Haggard et al., 2002), and is temporally “bound” in perception to one's own action. Moreover, the intensity of the sensory effect of a voluntary action is typically perceived as weaker than the same sensory event when it is externally generated (Shergill et al., 2003).

Such differences between self- and externally generated outcomes are not restricted to simple sensory perceptions: a large body of psychology literature has shown that people tend to overestimate their performance relative to others. For example, most people rate their driving ability (Svenson, 1981) or academic teaching performance (Cross, 1977) as above average. People also consistently show exaggerated optimism, unrealistically anticipating positive events in the future (Weinstein, 1980; Taylor, 1989). These “positive illusions” have been shown to span many cognitive and motor abilities, and have been linked to good mental health (Taylor et al., 2003b) and reduced physiological response to stress (Taylor et al., 2003a). Conversely, more accurate and less optimistic self-assessments and predictions of future outcomes are made by individuals with depression (Kuiper and MacDonald, 1982; Strunk et al., 2006).

It has been suggested that positive illusions arise from unrealistic predictions of performance, whereby optimistic expectations bias people's perception toward success (Dunning et al., 2003). Beliefs or expectations can indeed influence one's perception (Sterzer et al., 2008; Seriès and Seitz, 2013), for example, altering recognition accuracy (REF) (Sekuler and Ball, 1977) or the perceived intensity of pain and emotion (Colloca and Benedetti, 2005). The perception of the sensory consequences of one's own actions is modulated by beliefs about the cause of the sensory event, including the attribution of causality to either oneself or to an external agent (Desantis et al., 2011, 2012).

The perceptual changes induced by expectations or beliefs have been successfully formalized and quantified using Bayesian models. Here, we bring together research on the perception of action, on positive illusions and Bayesian models to investigate how people perceive the sensory consequences of self-generated actions compared to observed actions. We tested the hypothesis that a bias in people's perception of their performance results from unrealistic expectations of their own performance (Dunning et al., 2003).

In Bayesian models of perception, expectations of possible outcomes are represented by priors (Kersten et al., 2004; Körding and Wolpert, 2004). The brain generates perception by combining priors with the imperfect sensory information about the external world. The relative weighting given to priors and “sensory evidence” for this integration depends on their reliability, such that less variable signals are weighted more for perception. Perception of one's own actions that is biased toward success might have two main explanations in Bayesian models: (1) Stronger priors with narrower distributions (smaller SDs) have a greater effect on perception. For the perception of one's own actions, “optimistic” narrow priors distributed around the intended goal would lead to a stronger bias toward the perception of success during performance; (2) Less reliable visual evidence (“likelihood”)—that is, more sensory noise would lead to an increased reliance on priors and a greater perceptual bias. This latter hypothesis seems inadequate, since for perceiving the results of their own actions, people have multiple sources of sensory information, such as visual, proprioceptive and tactile signals. However, by fitting a set of models, we could experimentally test these alternate hypotheses.

Using novel visuomotor tasks, we first confirmed that the perception of the sensory consequences is biased toward success for one's own actions more than for observed actions. In Experiment 1 we show that this discrepancy could be explained by self priors that represented optimistic predictions of success, i.e., priors that are narrower than participant's true performance distribution. The exaggerated priors were unaffected by learning, but were related to individual differences in trait optimism. In contrast, when observing actions, participants' priors more accurately represent the true distribution of performance. In Experiment 2 and 3 we validated these results in different tasks and controlled for potential confounding factors.

## Experiment 1: main experiment

### Materials and methods

#### Experimental procedures

Participants in all experiments had no history of a neurological disorder and had normal or corrected-to-normal vision. They all provided written informed consent, and the study was approved by the Cambridge Research Ethics Committee. During all experiments, participants were seated 0.5 m from a 17 inch Dell CRT screen with 1024 × 768 resolution (25 pixels/cm) that refreshed at 85 Hz. All stimuli were displayed with Matlab Psychophysics toolbox (Brainard, 1997).

**Figure 1:**
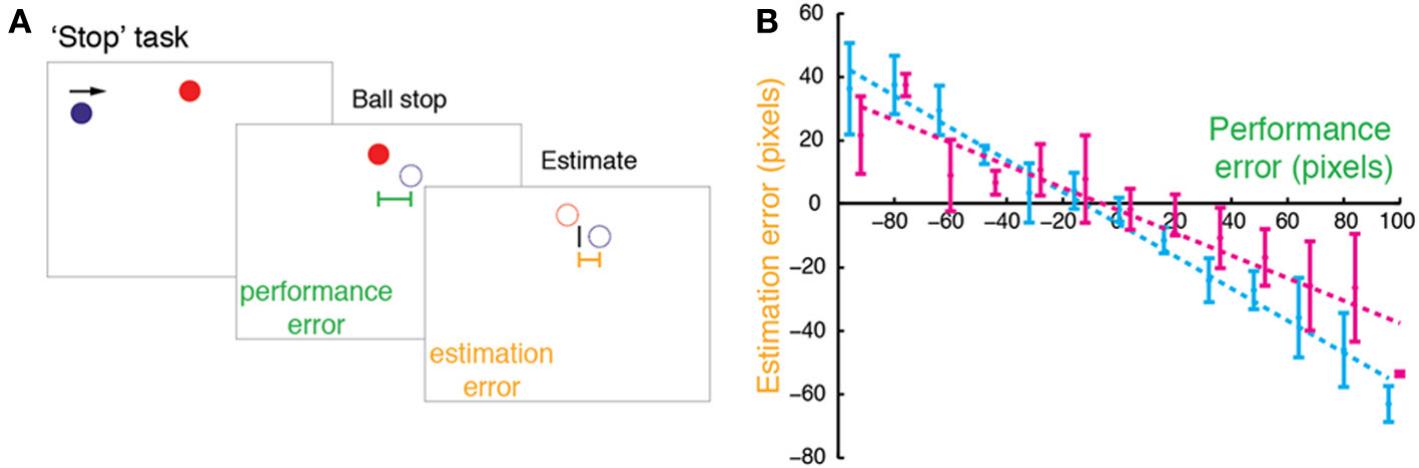
**“Stop” task in Experiment 1. (A)** In the Stop task, participants were asked to stop a moving blue ball when it was aligned with a stationary red target (Self condition) or watch the computer stopping the ball (Agent condition). After the ball was stopped, it vanished, and participants used a cursor to indicate the stopping point. **(B)** Estimation errors (difference between estimated and true position) plotted against performance errors (difference between the true position and the target) for a typical participant in the Stop task with regression line (dashed) and SDs (error bars) for Self (cyan) and Agent (magenta, offset by 3 pixels for illustration).

Twenty right-handed participants (11 females) aged 20–35 years (mean 26; *SD*: 4) took part in Experiment 1. In the experiment, participants performed the “Stop” task (Figure 1A), in which a red circle target (15 pixel radius) was horizontally centered close to the top of the screen. A blue ball (15 pixel radius) repeatedly swept horizontally in a rightward motion at 1200 pixels/s, passing just below the target, and reappearing at the opposite edge when it disappeared off the screen. The ball's starting position was randomized across trials (uniform distribution covering the horizontal extent of the screen). The participant's task was to stop the ball when it was exactly below the target by pressing a computer mouse button. Following the button press, the ball disappeared and 250 ms later the target disappeared. Using the computer mouse, participants then moved a rectangular cursor (3 pixels wide × 30 pixels high) to indicate the ball's stopping position (i.e., final position before vanishing). The cursor was constrained to move horizontally, and its starting position was drawn randomly from a uniform distribution ±150 pixels around the target.

In addition to performing the task themselves (Self condition), participants also observed the computer performing the task (Agent condition). In the Agent condition, participants pressed the mouse button when the computer stopped the ball. This allowed us to monitor levels of attention to the Agent condition throughout the experiment, and equate the Self and Agent conditions with regard to motor demands. Participants indicated the final position of the ball by moving the cursor as above. In the Agent condition, we replayed the final position and number of screen sweeps from each trial of the previous Self condition in a permuted order. However, to minimize awareness that these were replay trials, we limited the replay of outliers in terms of final position or number of sweeps: for each set of Self trials we calculated the mean and SD of the final positions and truncated the replay position ±2 SD of the corresponding Self condition. We also limited the number of screen sweeps to be between one and four.

During both Self and Agent conditions, participants were encouraged to fixate the target. In all experiments, Self and Agent conditions were blocked, and the Agent blocks were always performed after the Self blocks, so as to allow the computer to approximate each participant's performance (as described above). In Experiment 1, participants first completed one practice and two experimental blocks of each condition for acquiring “baseline” measures, which were also used for correlating with a questionnaire (see below). Subsequently, two “feedback” blocks were performed for each condition. In these blocks, feedback was given in order to explore the effect of learning: after each trial, the target, ball and estimation cursor were displayed for 1.5 s, indicating the veridical target, stopping, and estimation positions. The experiment finished with two blocks without feedback. Each block included 52 trials. At the end of the session, participants completed the Life Orientation Test (Scheier et al., 1994) for assessing their trait optimism on a scale from 0 to 24 (most optimistic).

#### Analyses

In each of the experiments, for each trial in a task with a target we calculated the estimation error (i.e., distance between estimated final position and true final position) and the performance error (i.e., distance between true final position and target). To examine any bias toward the target, we fit linear regressions of estimation error against performance error for each task and condition and for each participant. Trials with estimation times larger than 2 SD from the mean were excluded. In the Agent conditions, trials with reaction times greater than 2 SD from the mean were excluded in order to control for similar levels of attention to the task. On average, six trials were excluded in each experiment for each participant.

We inferred the priors used by participants for both Self and Agent conditions in the following way: On each trial, we assumed that for estimating the final position of the ball, participants can use sensory evidence *x*_evidence_ and a prior *p*(*x*). The variance of the prior could reflect the distribution of participant's performance or their exaggerated expectations of success. Bayes' rule could then be used to estimate the optimal final position of the ball, by finding the maximum of the posterior:

$$p\left(x|x_{\text{evidence}}\right)=p\left(x_{\text{evidence}}|x\right)\frac{p(x)}{p(x_{\text{evidence}})}$$

With *x*_prior_ corrupted by noise with variance σ^2^_prior_ and with *x*_evidence_ corrupted by noise with variance σ^2^_evidence_, the optimal estimate is then given by:

$$x_{\text{estimate}}=\text{w}*{\bar{x}}_{\text{prior}}+\left(1-\text{w}\right)*{\bar{x}}_{\text{evidence}}$$

where the weighting w is given by: $\text{w}=\frac{\sigma_{\text{evidence}}^{2}}{\sigma_{\text{prior}}^{2}+\sigma_{\text{evidence}}^{2}}$ (Ghahramani et al., 1997; Ernst and Banks, 2002). Assuming that the prior and the noise on the evidence are both Gaussian (assumptions which are often made for Bayesian model fitting), we used several Bayesian models to fit our data so as to maximize the likelihood, i.e., the probability of each participant's dataset. We then used the Bayesian Information Criterion (BIC; Schwarz, 1978) to select the model that best accounted for the data, using a threshold of BIC difference of 6 for “strong” evidence (Raftery, 1995). The model that was selected was used for all participants.

In Experiment 1, we assumed that *x*_prior_ is centered on the target and *x*_evidence_ is centered on the true stopping position of the ball (assuming no spatial shifts, which we test in Experiment 2). We fit the data with the following models: (1) The full model included a separate σ^2^_*prior*_ and σ^2^_evidence_ for Self and Agent conditions (4 parameters); (2) A reduced model constraining σ^2^_evidence_ to be identical across agents (3 parameters); (3) A reduced model constraining σ^2^_prior_ across agents (3 parameters). For the best model we found 95% confidence intervals on the parameters of the fit by bootstrapping for 5000 samples with replacements (Efron, 1988).

Using different priors across Self and Agent conditions allowed us to test the main hypothesis that people have exaggerated expectations of success for their own actions (a prior with a smaller standard deviation). The use of the different visual evidence allowed us to test whether the illusions of superiority might arise from sensory noise being specifically increased for one's own actions, which would result in an over-reliance on prior expectations and a greater perceptual bias.

### Results

In the main experiment we examined the difference between the perception of self-generated goal-directed actions and externally generated action effects. We quantified this in terms of Bayesian priors and explored whether these priors change with explicit feedback of the action effect as well as the possible relation between these priors and behavioral measures, such as positive illusions and task learning.

#### Difference between perception of self-generated and observed actions

We first measured the bias toward the target in the “Stop” task (Figure 1A), examining participants' estimation errors as a function of performance errors. Estimation errors were biased toward the position of the target—that is, participants consistently underestimated their performance errors (Figure 1B). Across participants, for both Self and Agent conditions this bias was graded and dependent on performance errors, as revealed by a consistent negative regression slope between estimation error and performance error, [slopes smaller than zero for both Self: *t*_(19)_ = −6.233, *p* < 0.001; and Agent: *t*_(19)_ = −2.907, *p* = 0.009]. This bias was greater for Self relative to Agent condition [*t*_(19)_ = −3.93, *p* < 0.001], indicating that participants' underestimation of performance errors was stronger for their own actions compared to observed actions.

#### Priors for self-generated and observed actions

The perceptual bias toward the target could be explained if participants use a prior that is centered on the target for estimating the stopping position of the ball. According to Bayesian theory, the stopping position could then be inferred as a weighted average of the sensory evidence (“noisy” visual signals about the stopping point) and the prior. Such a prior could represent the true statistical distribution of performance (Körding and Wolpert, 2004) or could be narrower based on optimistic predictions of success (i.e., people expect to be more precise than they really are; Dunning et al., 2003). Moreover, if the variability of the sensory evidence is high for one's own actions, there would be an increased reliance on the prior, and an increased perceptual bias. To investigate these hypotheses, we computed the priors and sensory evidence that participants used by fitting the data with a set of Bayesian models. Importantly, we approximated the performance errors in the Agent condition to each participant's own performance by replaying each participant's performance errors in a permuted order. Further, motor demands were equated in both conditions (see Materials and Methods).

We fit the data with the following Bayesian models: (1) a model with a different variance of visual evidence and priors for the Self and Agent conditions; (2) a reduced model with one parameter for the variance of visual evidence and two parameters for the variance of priors (one for each condition); (3) a reduced model with one prior and a different visual evidence for each condition. The results showed that model 1 was the preferred, compared to both model 2 (mean group BIC difference of 4; individually in 13/20 subjects) and model 3 (mean group BIC difference of 18; individually in 16/20 subjects). We thus report the SDs of visual evidence and priors, which were derived from the fit of model 1, and compare them across conditions.

**Figure 2:**
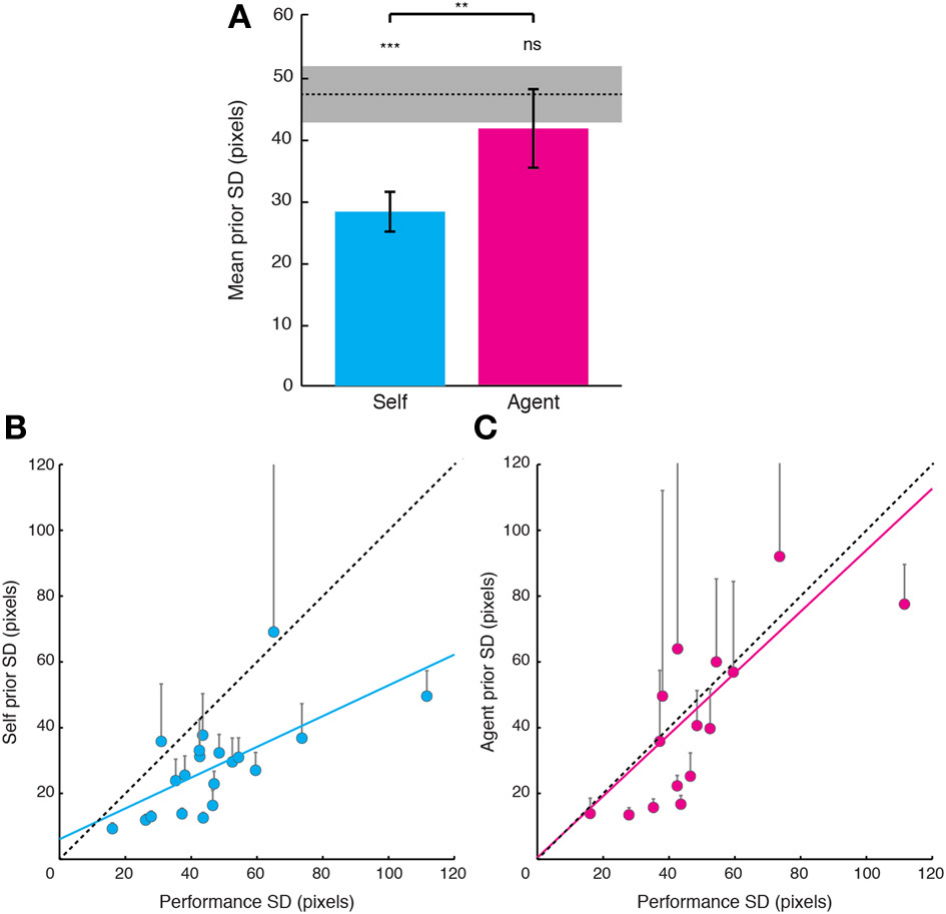
**Relations between priors for one's own actions, priors for observed actions and distributions of performance errors. (A)** Comparison of priors with performance SDs. Priors were narrower than performance distribution in the Self condition. By contrast, in the Agent condition, the SDs of priors were not different from performance in the 15 of 20 participants who showed Agent priors. Error bars indicate standard error of the mean. Shaded gray bar indicates the group standard error of the mean for performance errors. Significance levels indicated by ^***^p < 0.001, ^**^p < 0.01. **(B)** Self prior SDs plotted against performance error SDs. A significant correlation emerged across subjects (cyan regression line). Data of 18 of the 20 participants lie below the line of equality (black dashed line). Error bars indicate 95% confidence bounds. **(C)** Same as **(B)**, but for Agent priors against performance error SDs.

Visual evidence SD tended to be *smaller* in Self relative to Agent condition [*t*_(19)_ = −1.766, *p* = 0.094], suggesting that increased noise on visual evidence for Self relative to Agent condition could not account for the increased bias toward the target. We next compared the width of priors in the Self condition with the width of priors in the Agent condition and to performance SD (Figure 2A). The distributions of priors in the Self condition were narrower (smaller SDs) than performance distributions [*t*_(19)_ = −5.56, *p* < 0.001], suggesting that these priors are “optimistic” and do not simply reflect the statistics of performance. By contrast, the SDs of priors in the Agent condition were more variable. Of the 20 participants that were tested, five had “flat” priors, i.e., priors with very large SDs (>1E7). For the rest of the participants, the SDs of Agent priors did not differ from the SDs of performance errors [*t*_(14)_ = −1.67, *p* = 0.24], but were greater than the SDs of Self priors [*t*_(14)_ = 3.48, *p* = 0.008; Bonferroni corrected]. These Agent priors more closely represented the actual performance distribution compared to Self priors, as shown by a smaller absolute deviation from performance SD [*t*_(14)_ = −2.49, *p* = 0.03].

Next, we examined whether there was a consistent relation between the distributions of priors and performance. The SDs of Self priors were correlated with the SDs of performance errors (Figure 2B; *r* = 0.662, *p* = 0.002; slope of 0.47), suggesting that Self priors are scaled to individual performance. Agent priors were also strongly correlated with performance distribution (Figure 2C; *r* = 0.76, *p* = 0.001, slope of 0.848). Moreover, the SDs of Self and Agent priors also strongly correlated across participants (including participants with flat priors, using Spearman's rho = 0.62, *p* = 0.004), indicating that although participants varied widely, there was a consistent relation between the priors applied to one's own actions and those for observing actions. This relation might simply reflect the covariance of the SDs of both Self and Agent priors with the SDs of performance error. However, the correlation between the SDs of Self and Agent priors remained significant even after removing the effect of the covariance with performance (partial correlation; Spearman's rho = 0.52, *p* = 0.023).

#### Relation between exaggerated self priors and cognitive positive illusions

**Figure 3:**
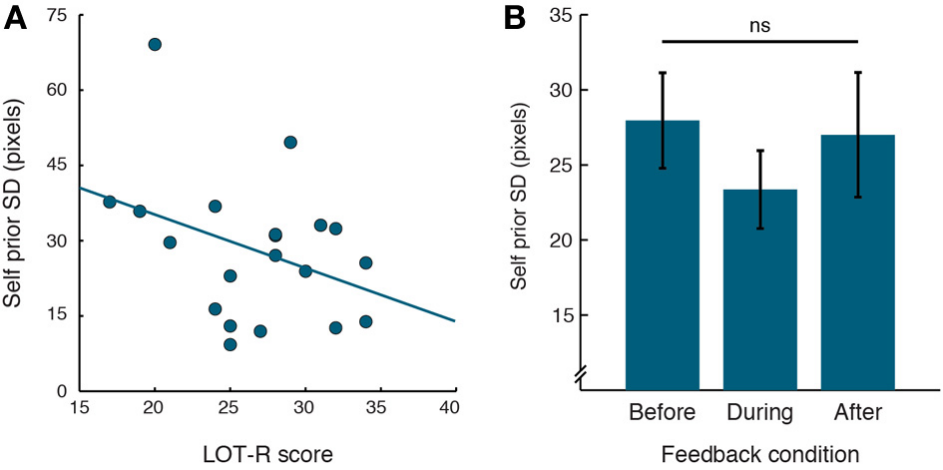
**Relation between Self priors and cognitive positive illusions. (A)** Correlation of Self priors with LOT-R, with linear regression line. **(B)** Self prior SDs plotted for the different feedback conditions. Prior SDs did not differ across feedback conditions. Error bars indicate standard error of the mean.

If the distributions of priors for one's own actions are linked to positive illusions of Self, then the width of priors might be related to individual differences in positivity such as trait optimism. We used the Life Orientation Test (LOT-R; Scheier et al., 1994) to measure variability in generalized optimism across participants. In a partial correlation analysis we accounted for variability in performance, in order to examine the relation between Self priors and LOT-R, independently of performance SD, which strongly correlated with the SDs of Self priors. The SDs of Self priors were significantly correlated with LOT-R scores across participants (Figure 3A; *r* = −0.474, *p* = 0.0402). This negative correlation indicates that participants with narrower Self priors tended to be more optimistic.

In order to further investigate the link between Self priors and cognitive positive illusions, we examined whether the exaggerated priors would be altered when people are informed of their overestimation of performance. If priors for one's own actions represent optimistic predictions of success that are linked to positive illusions, then these priors should be unchanged even when people receive feedback of their overestimation. To test this, we provided participants feedback for their own performance and estimation errors, by briefly displaying the veridical stopping position and estimated position of the ball as well as the target position after each trial. We examined the performance errors, estimation errors and model parameters before, during and after feedback, using repeated-measures ANOVAs (Greenhouse-Geisser corrected where appropriate). Feedback was a within-subject factor with three levels (before, during, or after feedback).

A main effect of feedback on performance error SD was found [*F*_(2, 38)_ = 6.645, *p* = 0.003]. *Post-hoc* Bonferroni corrected two-tailed *t*-tests revealed a reduction in performance error SD during, compared to before feedback [*t*_(19)_ = −3.342, *p* = 0.006]. Moreover, a strong trend for a reduction in performance error SD was found after compared to before the display of feedback [*t*_(19)_ = 2.395, *p* = 0.054]. Visual evidence SD was similarly affected by feedback [*F*_(1.43, 27.15)_ = 13.133, *p* < 0.001], with significant reductions during [*t*_(19)_ = 3.889, *p* = 0.002] and after feedback [*t*_(19)_ = 4.575, *p* < 0.001], compared to before the feedback display.

Examining the effect of feedback on Self priors, no main effect of feedback emerged [Figure 3B; *F*_(1.51, 28.62)_ = 1.304, *p* = 0.28]. Moreover, as priors were related to performance error (see above), we examined the difference between the two measures across feedback conditions. Again, no main effect of feedback emerged [*F*_(2, 38)_ = 0.935, *p* = 0.401]. Lastly, the effect of feedback on estimation errors, which according to our Bayesian models are driven by priors and visual evidence, was thus only trending for significance [*F*_(1.41, 26.74)_ = 2.987, *p* = 0.083].

As feedback trials were performed after no-feedback trials, these results could simply be related to the effect of practice. To control for this, we examined the effect of learning when no feedback was given during the first two blocks of trials. Here, participants' performance did not improve, as the SDs of performance errors were not reduced in the second compared to the first block [*t*_(19)_ = −1.07, *p* = 0.297]. Importantly, similar to the feedback manipulation above, the difference between the SDs of performance errors and priors remained unchanged [*t*_(19)_ = 1.11, *p* = 0.280].

#### Effect of priors on motor learning

If optimistic priors for one's own actions could lead to an underestimation of errors, it is possible that participants with narrow priors would show reduced motor learning and increased performance variability. The positive relation between priors and performance SDs across individuals (see above) does not support this, as it indicates that participants with narrow priors performed better in the task, rather than worse, with reduced performance variability. However, to examine this further, we looked at individual variability in motor learning.

For each participant we calculated the extent of improvement in the task as the difference between performance error SD during and before feedback (which showed a highly significant difference, above). Participants who were able to learn the task better thus showed a more negative difference. Although there was a negative relation between the extent of improvement in the task and width of Self priors, it was not significant across participants (*r* = −0.2597, *p* = 0.2689). We split the data into two equally sized groups according to the median of individual differences in Self priors. Although again participants with wider priors showed greater improvement in the variability of performance errors, there was no significant difference between the groups [*t*_(18)_ = −1.6006, *p* = 0.127].

### Experiment 1 discussion

We have shown that increased perceptual bias toward a target could be explained by the use of narrower Self priors compared to priors used for the observation of actions. The increased bias could not be explained by an increase in visual noise, as there was a trend toward reduced variability in sensory evidence for perceiving the consequences of one's own actions.

The variability of sensory evidence (the “likelihood”) for perceiving the outcomes of self-generated actions tended to be smaller compared to that of observed actions. This improved precision for self-generated actions might reflect the combination of sensory and predictive information available about one's own actions. In addition to visual input, these include tactile and proprioceptive information, and predictive signals based on the efference copy of motor commands, providing more precise information about the time of action and its consequent sensory effect. Additional information compensates for uncertainty in the visual stimuli. For example, although the estimation of the stopping point was continuous in our experiments, the ball stimulus moved on the screen in 14 pixel increments per 12 ms screen refresh. The visual uncertainty of 14 pixels could in principle be dealt with by extrapolating the ball's position according to the time at which the ball was stopped. With multisensory integration and predictive information during one's action, such extrapolation would be more precise in the Self condition.

Importantly, any differences in sensory precision that might occur between Self action and observation could not affect our estimates of the priors for the two conditions, as we allowed our Bayesian models to have different sensory noise for the two conditions. Estimation of the prior is thus independent of such noise. It is possible that multisensory and predictive signals would somehow lead people to enhance the reliability of the priors for their own actions compared to their observation priors. While we cannot rule out this account, it could not explain why Self priors are consistently narrower than performance, nor the correlation of the width of priors with optimism (see below).

We found that participants used priors that represent exaggerated expectations of success for their own actions (explaining the perceptual bias toward success), but priors that more accurately represent the true distribution of performance for observed actions. Could the fixed order, in which the Self condition preceded the Agent condition, account for the difference between Self and Agent priors? This fixed order was preferred over the conventional counterbalancing of conditions, in order to allow the computer to approximate participants' distribution of performance error in the Agent condition. The exaggerated narrow Self priors were still found even when only the Self condition was performed (see Experiment 3). Moreover, in the experiment, Self and Agent conditions were performed one after another—that is, although Self condition was performed first, one Self block was always followed by one Agent block, rather than a block design of performing all Self trials before Agent trials. An order effect would thus mean that the first Agent block could somehow affect all Agent blocks (as priors were calculated based on all blocks), however, this seems implausible.

People might in principle apply a different cost function when acting themselves and when observing actions, but we suggest that this is not sufficient to account for the difference between Self and Agent conditions, for the following reason. If the prior and sensory noise are Gaussian then the posterior must also be Gaussian. Many standard cost functions (e.g., mean squared error, absolute error, hit rate, or absolute error raised to any positive power) would identify the peak of the posterior distribution. This would also be true even if the prior and noise are not Gaussian, as long as the posterior is symmetric. Therefore, under their general assumptions, many alternate cost functions would not affect our estimate of the prior or sensory noise. This statement would not be true for atypical cost functions (asymmetric non-Gaussian), but such cost functions would require a special justification.

We observed a higher individual variability for Agent priors (vs. Self priors), with some participants showing “flat” Agent priors. This larger variability might be because people need more trials to learn the variance of performance distribution of observed stimuli. However, the number of trials provided in our experiment was not smaller than in a previous study, in which participants still demonstrated the use of priors (Berniker et al., 2010). Moreover, individual differences in assigning agency to the computer's “actions” might have also contributed to individual differences in Agent priors, and observing actual movements of another human agent might affect these observation priors (see General Discussion). Interestingly, although Self and Agent priors varied widely across participants, both priors for one's own actions and priors for observation were related to the individual's actual distribution of performance. In the General Discussion we discuss what might explain this consistent relation in the width of priors.

There was also a consistent relation between the width of Self priors and participants' individual differences in optimism, such that participants with narrower priors were more optimistic. Moreover, the results of the feedback conditions suggest that even when participants used feedback to improve in the task in terms of their performance errors, their priors did not significantly change and did not become more similar to the distribution of performance. These findings strongly support the cognitive illusions of superiority, to which we return in the General Discussion. Lastly, the data did not provide clear evidence for a relation between priors and motor learning, however, the nature of this experiment does not allow to rule out this possibility.

## Experiment 2: validation and control for potential confounding factors

### Materials and methods

#### Experimental procedures

Ten participants (five female) aged 19–27 years (mean: 24 ± 2) took part in Experiment 2. In Experiment 2, the Stop task was performed with both directions of ball motion in order to examine the effect of directionality and whether the priors were in fact centered on the target, as assumed in Experiment 1. The ball's direction of motion (leftward or rightward) was randomized across trials. The task was otherwise identical to the Stop task in Experiment 1.

**Figure 4:**
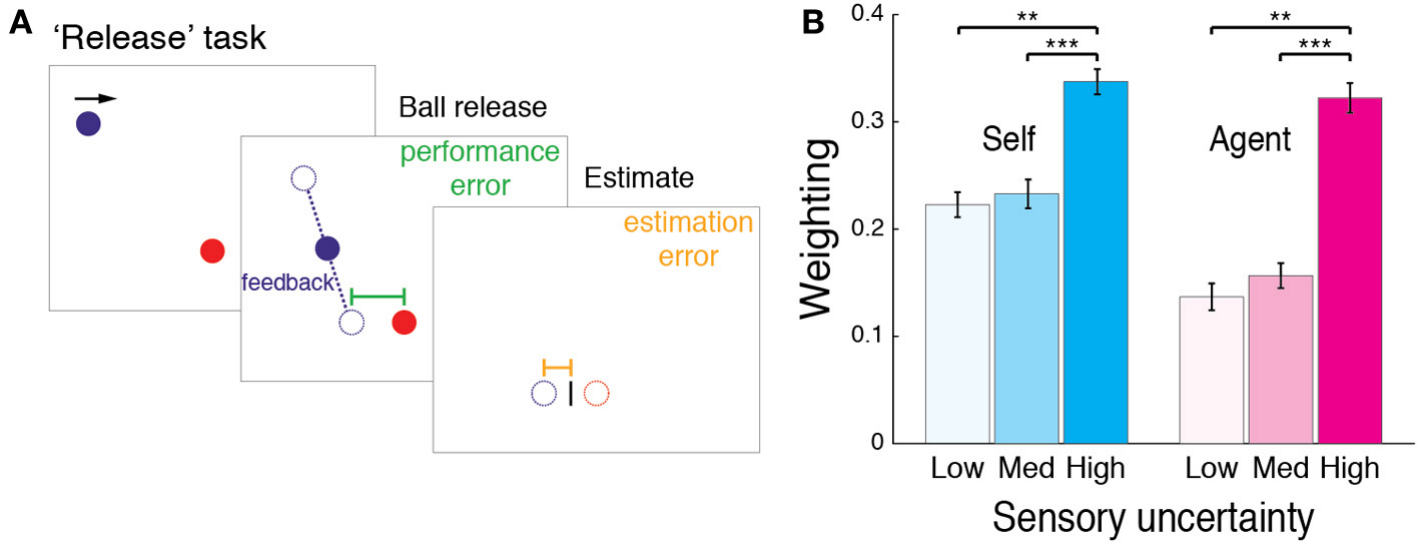
**“Release” task in Experiment 2. (A)** In the Release task, participants released the ball (Self) or observed it being released (Agent), so as to drop it onto a target. We varied the visual feedback in one of three ways: by only displaying the ball as it reached the target plane; displaying it briefly at the halfway point; or not displaying the ball after release. This manipulation provided low, medium, or high levels of uncertainty, before participants again estimated the ball's final position. **(B)** Across participant mean weighting (from regression slopes as in Figure 1B) for Release task. Error bars indicate SE. Significance levels of *post-hoc* tests indicated by ^***^p < 0.001, ^**^p < 0.01.

In the Stop task in Experiment 1, the ball vanished immediately after the action, preventing manipulation of sensory uncertainty. We therefore manipulated sensory uncertainty in a new “Release” task (Figure 4A). In the Release task, the target was at the bottom of the screen. The ball moved horizontally in a rightward motion across the top of the screen (490 pixels vertically above the target). The participant's task was to press the mouse button to release the ball so as to hit the target. On release, the ball fell to the target plane in 47 ms (4 screen refreshes) while moving horizontally at 2400 pixels/s. In other words, the ball did not drop straight, but had a constant lateral displacement which participants learned during practice trials, in which the full trajectory was displayed. Based on pilot experiments, the increase in horizontal speed was used in order to make the task more demanding.

Upon release, the ball vanished, and participants were asked to estimate the ball's final position, based on the “dropping” point (i.e., when the button was pressed), the lateral displacement of the descent and the different levels of feedback. We manipulated feedback in three ways: (1) displaying the ball for 24 ms as it reached the target plane; (2) displaying the ball for 24 ms when it was halfway to the target plane; or (3) not displaying the ball after release. These three feedback levels provided low, medium, and high levels of uncertainty with regard to the landing position. Feedback levels were pseudo-randomly interleaved across trials, such that overall, the same number of trials was carried out for each feedback level. Again, after the ball had vanished, participants moved a cursor to indicate the ball's final position (here the landing position). As in the Stop task, participants also performed an Agent condition, watching the computer performing the Release task; performed a reaction time task on when the ball was released; and indicated the ball's final position with the cursor.

To examine any spatial bias toward a certain point in the screen, we also included two “No Target” tasks, in which no visible target was displayed. Participants were asked to stop or release the ball at any time in its sweep in the Self condition or to watch the computer stopping or releasing the ball in the Agent condition. Again, participants indicated the final position of the ball.

Participants performed all tasks during two sessions, on separate days. Each session began with one practice block for each task and for the Self and Agent conditions. The practice blocks were identical to the experimental blocks for Stop and No Target Stop tasks. However, for Release and No Target Release tasks we displayed the ball throughout its descent to the target plane during practice trials, allowing participants to learn the properties of the descent. We note that although the descent trajectory makes the fall physically unrealistic, this does not affect our comparison of the Self and Agent conditions, as in both cases the ball descended in the same manner.

Each session included four experimental blocks for each task and condition in a pseudo-randomized order (with Agent following Self, as above), counterbalanced across participants. Each Stop and No Target Stop blocks included 32 trials, and each of the Release and No Target Release blocks included 48 trials. To control for eye movements, during the first experimental block in the second session, eye gaze position was recorded using an SMI iView X™ Hi-Speed eye tracker with a sampling rate of 500 Hz. Gaze events were detected using SMI BeGaze 3.0™.

#### Analyses

As the weighting *w* is given by:

$$\text{w}=\frac{\sigma_{\text{evidence}}^{2}}{\sigma_{\text{prior}}^{2}+\sigma_{\text{evidence}}^{2}}$$

(see Experiment 1), rewriting the second equation from Experiment 1 thus gives:

$$\underset{\text{estimation error}}{\underset{︸}{x_{\text{estimate}}-x_{\text{evidence}}}}=-w\underset{\text{performance error}}{\underset{︸}{\left(x_{\text{evidence}}-x_{\text{prior}}\right)}}$$

The magnitude of the negative slope of the linear regression fits therefore corresponds to the weighting *w*. We thus inferred the weighting for the Release task directly from the regression.

We next fit the two directions of motion separately in the Stop task, in order to examine any possible directional shifts in the means of sensory evidence and prior distributions. We fit a set of more complex models, now without assuming absence of shifts, wherein the optimal estimate is:

$$\begin{array}{l} x_{\text{estimate}}=\text{w}*({\bar{x}}_{\text{prior}}+shift_{\text{prior}})+\\ \;\left(1-\text{w}\right)*({\bar{x}}_{\text{evidence}}+shift_{\text{evidence}}) \end{array}$$

As the visual shifts were in the direction of motion in the No Target task (see Results), we assumed that *shift*_evidence_, related to a directionality effect of the ball motion would be opposite in the two directions of motion. In the full model, for each condition (Self and Agent), there was a separate *shift*_prior_, *shift*_evidence_, and σ^2^_prior_; The σ^2^_evidence_ was separate for each condition and direction of motion (10 parameters in total). Another model was fit, in which σ^2^_evidence_ was constrained across directions of motion (8 parameters).

### Results

#### Bayesian integration under different levels of sensory uncertainty

In Experiment 2, we aimed to replicate the results of Experiment 1 while controlling for several possible confounding factors, including the direction of motion of stimuli, spatial and directional shifts and differences in overt attention. We first examined the use of Bayesian integration under different levels of sensory uncertainty. According to Bayesian theory, the weighting of the prior should increase with increased sensory uncertainty (i.e., with more “noise” on sensory evidence). We manipulated the sensory evidence in the “Release” task (Figure 4A), which provided low, medium or high levels of uncertainty for estimating the ball's final position.

We inferred the weighting directly from the regression, as the magnitude of the negative slope corresponds to the relative weighting of the prior (see Materials and Methods). We compared the weighting across the different levels of sensory uncertainty, by submitting the data to a repeated-measures ANOVA, with sensory uncertainty (low, medium, high) as a within-subject factor. A significant main effect of uncertainty on the weighting emerged for both Self [*F*_(2, 18)_ = 12.611, *p* < 0.001] and Agent conditions [*F*_(2, 18)_ = 12.322, *p* < 0.001]. *Post-hoc* two-tailed (Bonferroni corrected) comparisons showed that as predicted by the Bayesian theory, the weighting was greater under high uncertainty feedback (Figure 4B), suggesting increased reliance on a prior for the perception of performance [Self: high-medium *t*_(9)_ = 5.63, *p* < 0.001 and high-low *t*_(9)_ = 3.83, *p* = 0.004; Agent: high-medium *t*_(9)_ = 5.3, *p* < 0.001 and high-low *t*_(9)_ = 3.59, *p* = 0.006].

#### Spatial and directional shifts

So far we have shown that participants' estimates were consistently biased toward the position of the target; a bias which we have explained through the use of priors. However, our results might be confounded by other visual biases or shifts arising from the nature of the stimuli. Particularly, participants might be generally biased toward a certain point on the screen; or their estimates might be shifted as a result of the ball motion. We examined these possible visual confounders.

**Figure 5:**
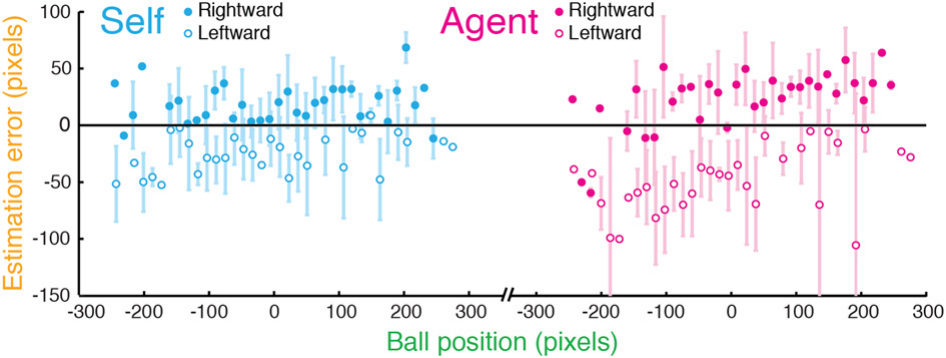
**“No Target” task in Experiment 2**. The data of a typical participant in the No Target Stop task, in which participants stopped the ball at any time in its sweep (Self) or watched it being stopped by the computer (Agent). Estimation errors are plotted against the ball position (distance from the center of the screen) for when the ball was sweeping in a rightward (filled circles) and a leftward (empty circles) motion. Error bars indicate SD. Estimation errors were not consistently shifted toward a certain point on the screen, but were typically in the direction of the ball's motion.

Across participants, we found no consistent spatial bias in estimation errors toward a certain point in the screen in a “No Target” task (Figure 5): regression slopes were not significantly different from zero for both Self and Agent [Self: *t*_(9)_ = 0.8856, *p* = 0.4; Agent: *t*_(9)_ = 1.556, *p* = 0.154]. Instead, estimation errors were consistently in the direction of ball motion, as previously found for the perception of moving stimuli (Freyd and Finke, 1984; Hubbard and Bharucha, 1988; Stork and Müsseler, 2004).

Since estimation errors were consistently shifted in the direction of ball motion in the absence of a target, we explored such possible directional shifts in the means of priors and sensory evidence. Participants performed the Stop task now with the two directions of ball motion. We fit a set of models to the two directions of motion separately, allowing for the means of priors and sensory evidence to vary (i.e., without constraining their means).

Based on both single-subject (10/10 participants) and group level model evidence (mean BIC difference of 17), the model that was selected had one SD of sensory evidence for the two directions of motion. The mean of sensory evidence was not different from zero across participants for both Self [*t*_(9)_ = 0.18, *ns*] and Agent [*t*_(9)_ = 1.58, *p* = 0.15] conditions. Moreover, the means of priors were not consistently different from the position of the target for both conditions [Self: *t*_(9)_ = −1.05, *p* = 0.32; Agent: *t*_(9)_ = 0.01, *ns*]. These results suggest that overall there were no consistent directional or spatial shifts in the Stop task—that is, the prior mean was not consistently shifted from the target position, and the sensory evidence mean was not consistently shifted from the true stopping position of the ball.

Crucially, the main pattern of results in this modified Stop task and in the Release task described above was consistent with the results of Experiment 1. That is, Self priors were narrower than both the distributions of performance (in 9/10 subjects for modified Stop task and 8/10 for Release task) and Agent priors (in 10/10 subjects for both modified Stop and Release tasks).

#### Eye gaze position in self and agent conditions

**Figure 6:**
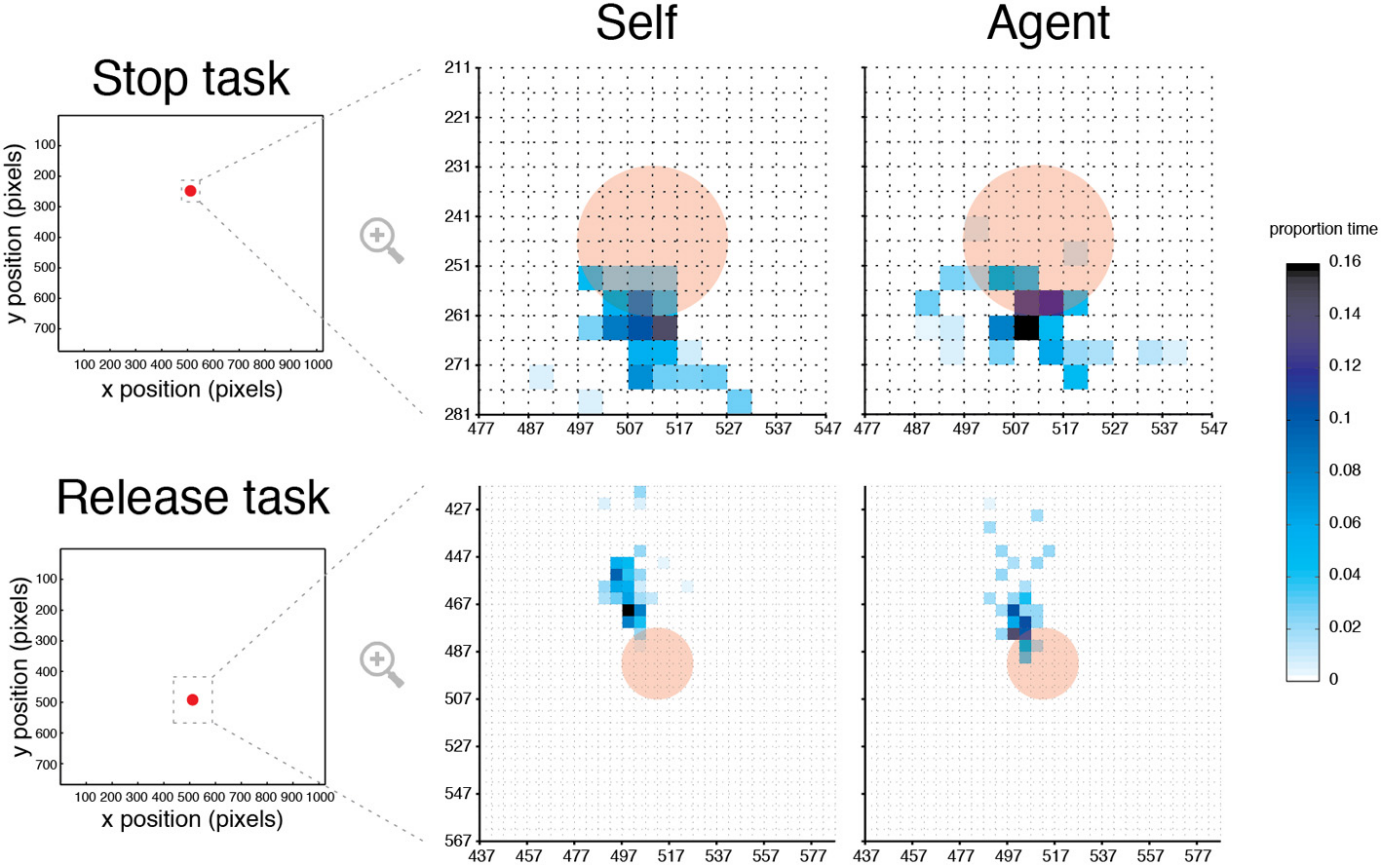
**Eye gaze position in Self and Agent conditions**. Heat maps illustrating the mean fixation time as a percentage of block duration for a typical participant in the Stop and Release tasks, superimposed on the target (opaque red). For illustration, an area of interest was selected so at to capture at least 98% of fixation duration data. For each task, this area was then “down-sampled” to 5 × 5 pixels datasets, where the color in each data point indicates the percentage of fixation duration.

Strategic or overt attention differences between Self and Agent conditions were examined by monitoring eye gaze position (Figure 6). An analysis of fixation proportion times across participants was performed with a circular area-of-interest. The selected size of this area was 6-times the target's size, which captured most of the variability in participants' fixation times, relative to other diameters. The analysis showed no effect of condition [*F*_(1, 9)_ = 0.62, *p* = 0.451] or condition × task interaction [*F*_(1, 9)_ = 0.423, *p* = 0.532]. These results suggest that participants fixated similarly around the target during both Self and Agent conditions.

### Experiment 2 discussion

The data of Experiment 2 confirmed that the results of Experiment 1 were likely to be due to differential Bayesian integration, with optimistic Self priors. In contrast, the results could not be explained by directional or general spatial shifts in sensory evidence or priors. The discrepancy between Self and Agent priors were unlikely to be due to differences in strategy or overt attention in terms of eye gaze position, which was similar across conditions. The exaggerated Self priors and more accurate observation priors persisted in these different control conditions of the experiment.

It is worth considering that the perception of feedback could differ when the sensory outcome results from self-generated and observed actions. For example, the visual feedback might be more informative when it is self-caused, as one could better predict the time at which the feedback is going to be displayed. However, the pattern of results across the different levels of uncertainty was consistent for both Self and Agent conditions: the weighting of priors was increased under high uncertainty, with a similar extent of increase across these conditions (see Figure 4B). Similarly, overt attention to the feedback was similar across conditions, as demonstrated by eye gaze position data. Notwithstanding this innate difference in the use of feedback in self-generated and externally caused actions, these results suggest that both Self and observed priors are integrated with sensory evidence in a similar uncertainty-dependent manner for generating a percept, as predicted by the Bayesian theory.

Importantly, when no target was displayed or specified in the No Target task, estimation errors were typically in the direction of motion, as described in the representational momentum phenomenon (Freyd and Finke, 1984). The spatial shifts were smaller when the ball was stopped by oneself relative to when it was stopped by the computer (see Figure 5). This pattern of results can be explained by the additional information one has about the sensory consequences of one's own action, as well as by the causality-induced spatial compression (Buehner and Humphreys, 2010). In the causality-induced compression, perceived causality relation can lead to a spatial contraction between a cause (e.g., an action) and its consequence (e.g., an ensuing sensory event). This might thereby reduce the spatial shifts of the representational momentum in the Self condition.

However, causality-induced compression cannot explain the consistent bias toward the target in the tasks in which a target is displayed or specified: the causality-induced space compression predicts that people's perceptual estimates would be shifted toward the point where an action was made—that is, where the ball was dropped or stopped, and hence a more accurate estimate, relative to observation. This prediction is not consistent with our results of a consistent bias toward the target and not toward the stopping or dropping point. Estimation errors were either to the right when the ball was stopped or dropped to the left of the target or to the left when the ball was stopped or dropped to the right of the target. This bias toward the target is thus qualitatively different to a causality-induced spatial compression.

## Experiment 3: different visual configurations

### Materials and methods

Six additional participants (four female) aged 21–27 years (mean: 24.5 ± 2) performed two control tasks to examine the effect of the visual configuration of targets. In one task, three red circles were displayed at the top of the screen, two of which were horizontally displaced ±100 pixels from the standard central target circle. In each condition, one of the three red circles was verbally specified as the target, and participants were asked to stop the moving ball when it was just below that target. This allowed us to assess whether estimation errors were related to the specified target, which varied between conditions, rather than simply the visual configuration, which was fixed across conditions.

In the second task, no visible target was displayed, and participants were asked to stop the ball when it was horizontally centered on the screen. This allowed us to examine estimation errors to an implicit target that had to be inferred, rather than the visibly specified targets in the other tasks.

Participants performed one practice and five experimental blocks for each task. The three conditions in the first task and the one condition in the second task were blocked, and were performed in a randomized order across participants. After a practice block, three experimental blocks were performed for each condition, with each block including 40 trials. For fitting the data, we used the same model as in Experiment 1.

### Results

In Experiment 3 we tested whether the bias toward the target and exaggerated Self priors persisted in different visual configurations in two control tasks. In the first task, three red circles were displayed at the top of the screen, and in each set of trials, one red circle was verbally specified as the target. Estimation errors were biased toward the specified target. As before, regression slopes were smaller than zero for all participants and for all targets. All priors were again smaller than performance SD, except for the case of one target for one participant.

In the second task, no visual target was displayed, and the target was only implied at the center of the screen. Estimation errors were again biased toward the center of the screen, with regression slopes always smaller than zero for all six participants. Prior SDs in five of the six participants were smaller than performance error SDs.

## General discussion

Our study finds that people's perception of their goal-directed actions is biased toward success. Using Bayesian models, which have accounted for different phenomena in visual perception (Kersten et al., 2004), we examined the larger perceptual bias for one's actions. We found that the bias can be explained by priors that are more narrowly distributed around the intended goal compared to the true distribution of performance. These narrow Self priors were related to participants' own performance and individual trait optimism, and were not influenced by learning. In contrast, priors used when observing actions more closely represented the actual distribution of performance.

The data from our control experiments showed that the exaggerated Self priors for one's actions could not be explained by a directionality effect or by a particular visual configuration of the stimuli. Moreover, although the Self and Agent conditions were different in several respects, we tested and controlled for several potential confounders. We showed that the discrepancy between priors for one's actions and for observed actions could not be explained by differences in overt attention strategy, observed performance distribution or motor demands.

### Mechanisms of self priors

In the sensorimotor system, priors are often obtained by learning to associate an action with its sensory effect over time (Körding and Wolpert, 2004; Synofzik et al., 2006). However, stable Self priors in our task were apparent in the first block of trials in Experiment 1. Moreover, the five participants who did not reliably obtain observation priors still all demonstrated Self priors, suggesting the acquisition of Self priors and observation priors does not rely on the same mechanism. By contrast, both Self priors and observation priors were strongly correlated, and both were related to the distribution of performance. About 44% of the variability in Self priors across participants was explained by performance variability (see Figure 2B), suggesting that even the unrealistic predictions of one's own success are *scaled* to one's actual performance (Ehrlinger and Dunning, 2003). Independently of their correlation with the distribution of performance, Self priors also correlated with observation priors. This relation suggests that across participants, some of the variability of both Self and observation priors might reflect individual differences in the general ability to form reliable predictions.

The relation between Self priors and individual differences in overall performance is intriguing, given the rapid acquisition and the independence of the optimistic predictions from feedback (see below). One explanation is that people may use their past experience of similar tasks to form an approximation of their performance even before starting a new task. Another possibility is that people form a fast approximation of their performance after only a few trials, which is in part supported by previous studies (Berniker et al., 2010). In both cases, after learning, people could update their measurement of performance and adjust their priors (as Self priors are scaled to performance), while keeping a similar level of exaggeration in these priors (as the difference between priors and performance remained similar with learning).

The exaggerated reliability of Self priors may result from an interaction between different prediction processes. For example, an efference copy of the motor command might provide the low-level sensorimotor prediction for motor adaptation (Wolpert and Ghahramani, 2000). These low-level predictions are acquired by learning the relation between an action and its effect over trials and can thereby reflect the performance in a task (Körding and Wolpert, 2004). On the other hand, high-level beliefs or expectations about the task could “exaggerate” the reliability of these priors, thereby linking the priors with illusory superiority and metacognitive measures, such as trait optimism. This low-level sensorimotor prediction and high-level perceptual expectations are likely to be supported by distinct mechanisms in the brain.

Our data do not address the question of how the brain represents the exaggeration of sensorimotor predictions. Cognitive positive illusions have been previously related to higher cognitive areas in the medial and lateral frontal cortex, particularly the anterior cingulate and amygdala of the mesolimbic and mesocortical pathways (Sharot et al., 2007; Beer and Hughes, 2010). It is possible that these areas exert a top-down modulation over sensorimotor areas, leading to the exaggeration of predictions and biased perception. The mechanism might also arise from reduced tracking of errors that call for a negative or pessimistic update of beliefs, mediated by the right inferior prefrontal gyrus (Sharot et al., 2011).

### Self priors and attribution of action

The difference between Self priors and observation priors builds upon previous work, showing that the perception of self-generated actions is different to the perception of external sensory events. A well-known example is the attenuation of the perceived intensity of one's action compared to when the same sensory stimulus is externally produced (Shergill et al., 2003). This sensorimotor attenuation relies on a prediction process (Bays et al., 2006), and has been suggested to facilitate the attribution of self-generated actions (Shergill et al., 2005).

Perceived success has also been demonstrated to contribute to the attribution of sensory consequences to one's own actions. For example, successful sensory outcomes tend to be attributed more to one's own actions than to an external source (Dewey et al., 2010). Similarly, people are less likely to detect an external perturbation to their actions, as long as their intended goal is achieved (Fourneret and Jeannerod, 1998). Moreover, the perceived temporal attraction of an action toward its sensory effect, which has been used as an implicit measure of agency (Haggard et al., 2002), is strengthened for actions that are associated with successful outcomes (Isham et al., 2011). Thus, the exaggerated expectations of success measured in Self priors and their consequent perceptual bias may enhance the self attribution of the sensory effects that result from one's own actions.

The sense of agency depends on an integration of multiple cues, including low-level sensorimotor signals and high-level expectations and perceptual processes (Synofzik et al., 2013). As noted above, Self priors might reflect this interaction of low-level predictions with high-level expectations, thereby capturing some of the key underlying mechanisms of the sense of agency. Future studies will shed light on how variability or changes in the experience of agency are reflected in the magnitude of exaggeration of Self priors for the perception of the consequences of one's actions.

### Stability of self priors with feedback

Veridical feedback of both performance and estimation did not affect the Self priors. Feedback improved the performance of participants in our task, both by reducing the variability in performance errors and by reducing the variability in visual noise for estimation. However, the estimated Self priors were unaffected by learning, as their distribution did not differ before, during or after the presentation of feedback. That is, even informing participants of their underestimation of performance errors did not change this behavior. Together with the association between the width of Self priors and trait optimism (see below), these findings speak directly to cognitive positive illusions, with which people persistently maintain a positively biased image of themselves and their future (Taylor et al., 1989). In other words, people fail to update their positive expectations of their own capabilities and of future events, even when confronted with reality (Armor and Taylor, 2002; Dunning et al., 2003). Our data suggest that this failure may result from a stable representation of unrealistically positive expectations in the brain.

### Effect of narrow self priors on sensorimotor learning

From the perspective of sensorimotor learning, it would seem sub-optimal for priors to over-represent the success of one's own performance, since learning critically depends on reliable and precise sensory feedback (Todorov and Jordan, 2002). In motor control theory, the comparison between the estimated state of the world and the desired state is crucial for updating the motor output in order to accurately achieve the desired goal (Wolpert and Ghahramani, 2000). Self priors leading to a diminished perception of one's own errors could impoverish this process by providing feedback that minimizes errors. Learning could therefore be impaired, resulting in increased long term performance errors.

High-level perceptual processes can, however, be independent of sensorimotor prediction processes. Whereas high-level expectations or beliefs about a task can change perceptual judgments (Sterzer et al., 2008), they may not influence sensorimotor processes, such as motor learning (Flanagan and Beltzner, 2000). These parallel systems may lead to a discrepancy between motor performance, which can still adapt properly to a changing environment, and illusory sensory perception (Flanagan and Beltzner, 2000). Consistent with this hypothesis, we did not find evidence for a negative effect of Self priors on motor learning. There was neither a consistent relation between the extent of improvement in our task and the width of priors, nor a difference in learning between participants with more and participants with less narrow priors (although the lack of statistical significance could also be related to a lack of power in the analyses). Further, the positive relation between performance and priors indicate that participants with narrow priors were superior in performance relative to participants with wider priors. The possible effect of narrow priors on learning remains to be investigated in future studies.

### Potential advantages of the narrow self priors

According to Bayesian models, priors are used to reduce variability in performance, while introducing a bias toward the prior mean. This trade-off between bias and variability is well captured in previous studies of Bayesian models of perception (Jazayeri and Shadlen, 2010) as well as in our study. Bayesian integration is said to “optimize” variability in performance, in the sense that the final estimate (posterior) has a lower variance than both the sensory evidence (“likelihood”) and prior. The importance of signal variability and “noise” are paramount for the normal physiology of the central nervous system (Faisal et al., 2008), and for the sensorimotor system in particular (Wolpert and Ghahramani, 2000). For example, in sensorimotor learning, motor noise during planning is taken into account for movement corrections, whose size in turn minimizes the final variability of movement (Van Beers, 2009).

The unique aspect of Self priors is that they may not require an initial learning in a new task. Although priors are normally acquired through experience over time, by learning the probability distribution of events over time (Berniker et al., 2010), Self priors are obtained faster and more reliably than observation priors. Indeed, all participants reliably showed Self priors for their own actions, whereas 5 of the 20 participants in Experiment 1 failed to demonstrate priors when observing actions. This fast formation of prior expectations may be particularly important when uncertainty about one's performance is high, for example during the performance of new motor tasks. Moreover, the fast formation of Self priors may reflect a commitment to the expectations of successful outcomes while alternatives are eliminated, promoting consistency in perception that leads to a more stable interpretation of the external world (Stocker and Simoncelli, 2008).

The underestimation of one's own errors (as a result of narrow Self priors) has been shown to support adaptive behavior under adverse circumstances in an unstable environment (Taylor and Brown, 1988). The ability to adapt appropriately to unexpected outcomes is crucial for learning and for the motivation to learn through exploration. Impairments in this ability may lead to “helplessness,” the belief that one's actions can no longer influence the environment, and the consequent passive behavior observed in individuals with, or at risk of, depression (Seligman, 1972). In Bayesian terms, helplessness has been formalized as prior beliefs which underestimate one's control over the environment (Huys and Dayan, 2009). Low-control priors in individuals with depression have detrimental effects on performance in that they diminish exploratory behavior, as rewards become less exploitable and punishments less avoidable (Huys and Dayan, 2009). Priors that overestimate one's control can therefore be more adaptive, supporting psychological well-being.

Similarly, wider (less “optimistic”) priors for one's action may be used in people with depression who exhibit a “depressive realism” (Alloy and Abramson, 1988)—that is, a more accurate perception of their own actions, with a smaller bias toward success (Alloy and Abramson, 1979; Kuiper and MacDonald, 1982). Consistent with this hypothesis, we found an association between the width of Self priors and trait optimism, suggesting that people with narrower Self priors tend to be more optimistic. That is, people who perceive the results of their actions to be closer to their goal in the present, expect more positive outcomes in the future. Taken together, narrow Self priors for one's own actions may have a significant contribution to adaptive behavior and psychological resilience.

Other advantages of narrow Self priors are suggested by evolutionary models, wherein the apparent short term sub-optimality in the perception of one's errors becomes advantageous in the longer term. For example, when evaluating oneself under uncertainty, if the cost of making false positive estimations is smaller than the cost of false negative estimations, evolution would favor overestimation (Haselton and Buss, 2000; Haselton and Nettle, 2006). Other evolutionary models have similarly shown that individuals who overestimate their capabilities tend to claim resources they could not otherwise win, provided that the benefits from the reward at conflict is sufficiently large relative to the cost of competition (Johnson and Fowler, 2011). Perception of enhanced performance also reduces the risk of giving up on resources individuals can surely win if it came to a conflict (Johnson and Fowler, 2011), and increases the accumulation of resources, by contesting in a larger number of conflicts (Johnson et al., 2011). Self-deceptive superiority is also important in deterring opponents, thereby increasing the probability of successfully claiming resources (Wrangham, 1999; Trivers, 2000). Finally, in Bayesian models of control, prior beliefs which underestimate, but not those that overestimate one's control over the environment, have detrimental effects on performance (Huys and Dayan, 2009).

### Action observation priors

Unlike the exaggerated Self priors for one's own actions, priors used for observing actions were more likely to represent the learned distribution of sensory stimuli according to their real probabilities. This was supported by the findings that the variance of these observation priors was highly correlated with, and not different from, the true distribution of the observed performance. Importantly, compared to Self priors, the absolute deviation of observation priors from performance was smaller. This accurate acquisition of the variance of observation priors and the learning of the distribution of stimuli was relatively fast in our study (Berniker et al., 2010), as these priors were calculated from about 100 experimental trials, following 50 practice trials.

Participants in our study showed high between-subject variability in these observation priors, and five participants showed “flat” priors, suggesting they did not form reliable predictions for perceiving the sensory consequences of observed actions. The high variability in priors for observation could reflect individual differences in the ability to generate sensorimotor predictions for external events. Moreover, the high variability could express individual differences in the attribution of an agent characteristic to the observed computer's actions and recognition of their intention or goal (Kilner, 2011). For example, participants who attribute more agency traits to the computer's actions might show narrower observation priors that are more similar to those used for one's actions.

Observation priors have been suggested to support the prediction and understanding of observed actions through the recruitment of the motor system (Gallese et al., 1996; Kilner, 2011). The understanding of an observed action plays an important role in social interactions as well as in motor learning and skill acquisition (Mattar and Gribble, 2005). Although we generalize our results in the Agent condition to observed actions, the observation priors in our study were not based on the observation of another human agent performing the task. These priors might thus be mainly based on the predictions of observed stimuli in general, and be less influenced by the social cognitive factors that typically accompany the observation of another human agent act.

Inferences of agency, however, do not require the explicit observation of a human agent (Castelli et al., 2002). Moreover, an action is not synonymous with a body part or movement of that body part, but is the means of achieving a goal (Passingham, 1993). Our task design thus still allows for the fundamental interpretation that differences in agency could lead to the use of different priors for the perception of the sensory consequences of actions.

## Conclusions

In conclusion, our results show that people's perception of the sensory consequences of their own actions is biased toward success, which can be explained by priors that represent optimistic predictions of performance. More research is required to establish the specific neural substrates of Self priors, their benefits in health, and how they might be altered in neuropsychiatric diseases. Nonetheless, our study shows that Self priors can bind the sensory consequences of our own actions with our intended goal, explaining how it is that when acting we tend to see what we want to see.

### Conflict of interest statement

The Associate Editor declares that, despite being affiliated to the same institution as authors Noham Wolpe, Daniel M. Wolpert, and James B. Rowe, the review process was handled objectively and no conflict of interest exists. The authors declare that the research was conducted in the absence of any commercial or financial relationships that could be construed as a potential conflict of interest.

## References

[B1] Alloy L. B. Abramson L. Y. (1979). Judgment of contingency in depressed and nondepressed students: sadder but wiser? J. Exp. Psychol. Gen. 108, 441–485 10.1037/0096-3445.108.4.441 528910

[B2] Alloy L. B. Abramson L. Y. (1988). “Depressive realism: four theoretical perspectives,” in Cognitive Processes in Depression, ed Alloy L. B. (New York, NY: Guilford Press), 223–265

[B3] Armor D. A. Taylor S. E. (2002). “When predictions fail: the dilemma of unrealistic optimism,” in Heuristics and Biases: The Psychology of Intuitive Judgment, eds Gilovich T. Griffin D. Kahneman D. (New York, NY: Cambridge University Press), 334–348

[B4] Bays P. M. Flanagan J. R. Wolpert D. M. (2006). Attenuation of self-generated tactile sensations is predictive, not postdictive. PLoS Biol. 4:e28 10.1371/journal.pbio.0040028 16402860 PMC1334241

[B5] Beer J. S. Hughes B. L. (2010). Neural systems of social comparison and the “above-average” effect. Neuroimage 49, 2671–2679 10.1016/j.neuroimage.2009.10.075 19883771

[B6] Berniker M. Voss M. Kording K. (2010). Learning priors for Bayesian computations in the nervous system. PLoS ONE 5:e12686 10.1371/journal.pone.0012686 20844766 PMC2937037

[B7] Brainard D. H. (1997). The psychophysics toolbox. Spat. Vis. 10, 433–436 10.1163/156856897X00357 9176952

[B8] Buehner M. J. Humphreys G. R. (2010). Causal contraction: spatial binding in the perception of collision events. Psychol. Sci. 21, 44–48 10.1177/0956797609354735 20424021

[B9] Castelli F. Frith C. Happé F. Frith U. (2002). Autism, Asperger syndrome and brain mechanisms for the attribution of mental states to animated shapes. Brain 125, 1839–1849 10.1093/brain/awf189 12135974

[B10] Colloca L. Benedetti F. (2005). Placebos and painkillers: is mind as real as matter? Nat. Rev. Neurosci. 6, 545–552 10.1038/nrn1705 15995725

[B11] Cross K. P. (1977). Not can, but will college teaching be improved? New Dir. High. Educ. 1977, 1–15 10.1002/he.36919771703

[B12] Desantis A. Roussel C. Waszak F. (2011). On the influence of causal beliefs on the feeling of agency. Conscious. Cogn. 20, 1211–1220 10.1016/j.concog.2011.02.012 21396831

[B13] Desantis A. Weiss C. Schütz-Bosbach S. Waszak F. (2012). Believing and perceiving: authorship belief modulates sensory attenuation. PLoS ONE 7:e37959 10.1371/journal.pone.0037959 22666424 PMC3362539

[B14] Dewey J. A. Seiffert A. E. Carr T. H. (2010). Taking credit for success: the phenomenology of control in a goal-directed task. Conscious. Cogn. 19, 48–62 10.1016/j.concog.2009.09.007 19833535

[B15] Dunning D. Johnson K. Ehrlinger J. Kruger J. (2003). Why people fail to recognize their own incompetence. Curr. Dir. Psychol. Sci. 12, 83–87 10.1111/1467-8721.01235

[B16] Efron B. (1988). The Jackknife, the Bootstrap and other Resampling Plans. Philadelphia, PA: Society for Industrial and Applied Mathematics

[B17] Ehrlinger J. Dunning D. (2003). How chronic self-views influence (and potentially mislead) estimates of performance. J. Pers. Soc. Psychol. 84, 5–17 10.1037/0022-3514.84.1.5 12518967

[B18] Ernst M. O. Banks M. S. (2002). Humans integrate visual and haptic information in a statistically optimal fashion. Nature 415, 429–433 10.1038/415429a 11807554

[B19] Faisal A. A. Selen L. P. J. Wolpert D. M. (2008). Noise in the nervous system. Nat. Rev. Neurosci. 9, 292–303 10.1038/nrn2258 18319728 PMC2631351

[B20] Flanagan J. R. Beltzner M. A. (2000). Independence of perceptual and sensorimotor predictions in the size-weight illusion. Nat. Neurosci. 3, 737–741 10.1038/76701 10862708

[B21] Fourneret P. Jeannerod M. (1998). Limited conscious monitoring of motor performance in normal subjects. Neuropsychologia 36, 1133–1140 10.1016/S0028-3932(98)00006-2 9842759

[B22] Freyd J. J. Finke R. A. (1984). Representational momentum. J. Exp. Psychol. Learn. Mem. Cogn. 10, 126–132 10.1037/0278-7393.10.1.12610660297

[B23] Gallese V. Fadiga L. Fogassi L. Rizzolatti G. (1996). Action recognition in the premotor cortex. Brain 119, 593–609 10.1093/brain/119.2.593 8800951

[B24] Ghahramani Z. Wolpert D. M. Jordan M. I. (1997). “Computational models of sensorimotor integration,” in Advances in Psychology: Self-Organization, Computational Maps and Motor Control, eds Morasso P. Sanguineti V. (Amsterdam: Elsevier), 117–147

[B25] Haggard P. Clark S. Kalogeras J. (2002). Voluntary action and conscious awareness. Nat. Neurosci. 5, 382–385 10.1038/nn827 11896397

[B26] Haselton M. G. Buss D. M. (2000). Error management theory: a new perspective on biases in cross-sex mind reading. J. Pers. Soc. Psychol. 78, 81–91 10.1037/0022-3514.78.1.81 10653507

[B27] Haselton M. G. Nettle D. (2006). The paranoid optimist: an integrative evolutionary model of cognitive biases. Pers. Soc. Psychol. Rev. 10, 47–66 10.1207/s15327957pspr1001_3 16430328

[B28] Hubbard T. L. Bharucha J. J. (1988). Judged displacement in apparent vertical and horizontal motion. Percept. Psychophys. 44, 211–221 10.3758/BF03206290 3174353

[B29] Huys Q. J. M. Dayan P. (2009). A Bayesian formulation of behavioral control. Cognition 113, 314–328 10.1016/j.cognition.2009.01.008 19285311

[B30] Isham E. A. Banks W. P. Ekstrom A. D. Stern J. A. (2011). Deceived and distorted: game outcome retrospectively determines the reported time of action. J. Exp. Psychol. Hum. Percept. Perform. 37, 1458–1469 10.1037/a0023111 21500944

[B31] Jazayeri M. Shadlen M. N. (2010). Temporal context calibrates interval timing. Nat. Neurosci. 13, 1020–1026 10.1038/nn.2590 20581842 PMC2916084

[B32] Johnson D. D. P. Fowler J. H. (2011). The evolution of overconfidence. Nature 477, 317–320 10.1038/nature10384 21921915

[B33] Johnson D. D. P. Weidmann N. B. Cederman L.-E. (2011). Fortune favours the bold: an agent-based model reveals adaptive advantages of overconfidence in war. PLoS ONE 6:e20851 10.1371/journal.pone.0020851 21731627 PMC3123293

[B34] Kersten D. Mamassian P. Yuille A. (2004). Object perception as Bayesian inference. Annu. Rev. Psychol. 55, 271–304 10.1146/annurev.psych.55.090902.142005 14744217

[B35] Kilner J. M. (2011). More than one pathway to action understanding. Trends Cogn. Sci. 15, 352–357 10.1016/j.tics.2011.06.005 21775191 PMC3389781

[B36] Körding K. P. Wolpert D. M. (2004). Bayesian integration in sensorimotor learning. Nature 427, 244–247 10.1038/nature02169 14724638

[B37] Kuiper N. A. MacDonald M. R. (1982). Self and other perception in mild depressives. Soc. Cogn. 1, 223–239 10.1521/soco.1982.1.3.223

[B38] Mattar A. A. G. Gribble P. L. (2005). Motor learning by observing. Neuron 46, 153–160 10.1016/j.neuron.2005.02.009 15820701

[B39] Passingham R. E. (1993). The Frontal Lobes and Voluntary Action. Oxford: Oxford University Press

[B40] Raftery A. E. (1995). Bayesian model selection in social research. Sociol. Methodol. 25, 111–163 10.2307/271063

[B41] Scheier M. F. Carver C. S. Bridges M. W. (1994). Distinguishing optimism from neuroticism (and trait anxiety, self-mastery, and self-esteem): a reevaluation of the life orientation test. J. Pers. Soc. Psychol. 67, 1063–1078 10.1037/0022-3514.67.6.1063 7815302

[B42] Schwarz G. (1978). Estimating the dimension of a model. Ann. Stat. 6, 461–464 10.1214/aos/1176344136

[B43] Sekuler R. Ball K. (1977). Mental set alters visibility of moving targets. Science 198, 60–62 10.1126/science.897682 897682

[B44] Seligman M. E. (1972). Learned helplessness. Annu. Rev. Med. 23, 407–412 10.1146/annurev.me.23.020172.002203 4566487

[B45] Seriès P. Seitz A. R. (2013). Learning what to expect (in visual perception). Front. Hum. Neurosci. 7:668 10.3389/fnhum.2013.00668 24187536 PMC3807544

[B46] Sharot T. Korn C. W. Dolan R. J. (2011). How unrealistic optimism is maintained in the face of reality. Nat. Neurosci. 14, 1475–1479 10.1038/nn.2949 21983684 PMC3204264

[B47] Sharot T. Riccardi A. M. Raio C. M. Phelps E. A. (2007). Neural mechanisms mediating optimism bias. Nature 450, 102–105 10.1038/nature06280 17960136

[B48] Shergill S. S. Bays P. M. Frith C. D. Wolpert D. M. (2003). Two eyes for an eye: the neuroscience of force escalation. Science 301, 187 10.1126/science.1085327 12855800

[B49] Shergill S. S. Samson G. Bays P. M. Frith C. D. Wolpert D. M. (2005). Evidence for sensory prediction deficits in schizophrenia. Am. J. Psychiatry 162, 2384–2386 10.1176/appi.ajp.162.12.2384 16330607

[B50] Sterzer P. Frith C. Petrovic P. (2008). Believing is seeing: expectations alter visual awareness. Curr. Biol. 18, R697–R698 10.1016/j.cub.2008.06.021 18727901

[B51] Stocker A. Simoncelli E. (2008). A Bayesian model of conditioned perception. Adv. Neural Inf. Process. Syst. 20, 1409–1416 Available online at: http://papers.nips.cc/book/advances-in-neural-information-processing-systems-20-2007 PMC4199208 25328364

[B52] Stork S. Müsseler J. (2004). Perceived localizations and eye movements with action−generated and computer−generated vanishing points of moving stimuli. Vis. Cogn. 11, 299–314 10.1080/13506280344000365

[B53] Strunk D. R. Lopez H. DeRubeis R. J. (2006). Depressive symptoms are associated with unrealistic negative predictions of future life events. Behav. Res. Ther. 44, 861–882 10.1016/j.brat.2005.07.001 16126162

[B54] Svenson O. (1981). Are we all less risky and more skillful than our fellow drivers? Acta Psychol. (Amst). 47, 143–148 10.1016/0001-6918(81)90005-6

[B55] Synofzik M. Thier P. Lindner A. (2006). Internalizing agency of self-action: perception of one's own hand movements depends on an adaptable prediction about the sensory action outcome. J. Neurophysiol. 96, 1592–1601 10.1152/jn.00104.2006 16738220

[B56] Synofzik M. Vosgerau G. Voss M. (2013). The experience of agency: an interplay between prediction and postdiction. Front. Psychol. 4:127 10.3389/fpsyg.2013.00127 23508565 PMC3597983

[B57] Taylor S. E. (1989). Positive Illusions: Creative Self-Deception and the Healthy Mind. New York, NY: Basic Books

[B58] Taylor S. E. Brown J. D. (1988). Illusion and well-being: a social psychological perspective on mental health. Psychol. Bull. 103, 193–210 10.1037/0033-2909.103.2.193 3283814

[B59] Taylor S. E. Collins R. L. Skokan L. A. Aspinwall L. G. (1989). Maintaining positive illusions in the face of negative information: getting the facts without letting them get to you. J. Soc. Clin. Psychol. 8, 114–129 10.1521/jscp.1989.8.2.114

[B60] Taylor S. E. Lerner J. S. Sherman D. K. Sage R. M. McDowell N. K. (2003a). Are self-enhancing cognitions associated with healthy or unhealthy biological profiles? J. Pers. Soc. Psychol. 85, 605–615 10.1037/0022-3514.85.4.605 14561115

[B61] Taylor S. E. Lerner J. S. Sherman D. K. Sage R. M. McDowell N. K. (2003b). Portrait of the self-enhancer: well adjusted and well liked or maladjusted and friendless? J. Pers. Soc. Psychol. 84, 165–176 10.1037/0022-3514.84.1.165 12518977

[B62] Todorov E. Jordan M. I. (2002). Optimal feedback control as a theory of motor coordination. Nat. Neurosci. 5, 1226–1235 10.1038/nn963 12404008

[B63] Trivers R. (2000). The elements of a scientific theory of self-deception. Ann. N.Y. Acad. Sci. 907, 114–131 10.1111/j.1749-6632.2000.tb06619.x 10818624

[B64] Van Beers R. J. (2009). Motor learning is optimally tuned to the properties of motor noise. Neuron 63, 406–417 10.1016/j.neuron.2009.06.025 19679079

[B65] Weinstein N. D. (1980). Unrealistic optimism about future life events. J. Pers. Soc. Psychol. 39, 806–820 10.1037/0022-3514.39.5.806

[B66] Wolpert D. M. Ghahramani Z. (2000). Computational principles of movement neuroscience. Nat. Neurosci. 3, 1212–1217 10.1038/81497 11127840

[B67] Wrangham R. (1999). Is military incompetence adaptive? Evol. Hum. Behav. 20, 3–17 10.1016/S1090-5138(98)00040-3

